# *BrLAS*, a GRAS Transcription Factor From *Brassica rapa*, Is Involved in Drought Stress Tolerance in Transgenic Arabidopsis

**DOI:** 10.3389/fpls.2018.01792

**Published:** 2018-12-06

**Authors:** Pan Li, Bin Zhang, Tongbing Su, Peirong Li, Xiaoyun Xin, Weihong Wang, Xiuyun Zhao, Yangjun Yu, Deshuang Zhang, Shuancang Yu, Fenglan Zhang

**Affiliations:** ^1^Beijing Vegetable Research Center (BVRC), Beijing Academy of Agriculture and Forestry Science (BAAFS), Beijing, China; ^2^Beijing Key Laboratory of Vegetable Germplasm Improvement, Beijing, China; ^3^Key Laboratory of Biology and Genetic Improvement of Horticultural Crops (North China), Ministry of Agriculture, Beijing, China

**Keywords:** *Brassica rapa*, GRAS transcription factor, *BrLAS*, drought tolerance, ABA sensitivity

## Abstract

GRAS proteins belong to a plant-specific transcription factor family and play roles in diverse physiological processes and environmental signals. In this study, we identified and characterized a GRAS transcription factor gene in *Brassica rapa, BrLAS*, an ortholog of Arabidopsis *AtLAS*. *BrLAS* was primarily expressed in the roots and axillary meristems, and localized exclusively in the nucleus of *B. rapa* protoplast cells. qRT-PCR analysis indicated that *BrLAS* was upregulated by exogenous abscisic acid (ABA) and abiotic stress treatment [polyethylene glycol (PEG), NaCl, and H_2_O_2_]. *BrLAS*-overexpressing Arabidopsis plants exhibited pleiotropic characteristics, including morphological changes, delayed bolting and flowering time, reduced fertility and delayed senescence. Transgenic plants also displayed significantly enhanced drought resistance with decreased accumulation of ROS and increased antioxidant enzyme activity under drought treatment compared with the wild-type. Increased sensitivity to exogenous ABA was also observed in the transgenic plants. qRT-PCR analysis further showed that expression of several genes involved in stress responses and associated with leaf senescence were also modified. These findings suggest that *BrLAS* encodes a stress-responsive GRASs transcription factor that positively regulates drought stress tolerance, suggesting a role in breeding programs aimed at improving drought tolerance in plants.

## Introduction

Transcription factors are known to participate in various growth and development processes in higher plants. GRAS proteins belong to a plant-specific protein family, named after the first three members identified, GAI, RGA, and SCR (Peng et al., 1997; Pysh et al., 1999; Bolle, 2004). GRAS proteins typically consist of 400–770 amino acids and possess a highly conserved homologous sequence at the C-terminus, which includes five conserved motifs, LHR I, VHIID, LHR II, PFYRE, and SAW (Pysh et al., 1999; Bolle, 2004; Lee et al., 2008). In contrast, the N-terminus is highly variable in both length and sequence. Studies have shown that the N-terminus of GRAS proteins can form a unique stretch region, perhaps contributing to the functional specificity of each member (Tian et al., 2004; Sun et al., 2011).

Members of the GRAS family reportedly possess a variety of functions, playing important roles in diverse physiological processes, such as gibberellin signal transduction, root radial patterning, axillary meristems development, phytochrome signaling, male gametogenesis and detoxification (Dill et al., 2001; Greb et al., 2003; Bolle, 2004; Lee et al., 2008). Two rice GRAS genes, *OsCIGR1* and *OsCIGR2*, show sensitivity to *N*-acetylchitooligosaccharide elicitor and are induced by phytoactive gibberellins (Day et al., 2004), while *AtRGA* and *AtGAI* negatively regulate a series of GA signal transduction pathways (Peng et al., 1997; Silverstone et al., 1998) and are major inhibitors of Arabidopsis vegetative growth (Tyler et al., 2004). Meanwhile, *AtSCR* and *AtSHR* participate in radial organization of the roots by forming a SCR/SHR complex (Cui et al., 2007), while *AtSCL3* plays an essential role in gibberellin-promoted cell elongation during root development via the formation of a regulation network with the DELLA protein and SHR/SCR complex (Heo et al., 2011). The PAT1 sub-branch in Arabidopsis, which consists of four GRAS proteins, PAT1, SCL5, SCL13, and SCL21, mediates phytochrome signaling pathways (Bolle et al., 2000; Torres-Galea et al., 2006, 2013). *LiSCl*, a GRAS gene isolated from *Lilium longiflorum*, participates in microsporogenesis of lily pollen by affecting the transcriptional activity of meiosis-related genes (Morohashi et al., 2003). The LS subfamily of GRAS proteins plays important regulatory roles in axillary meristem development. Analysis of the lateral suppressor (*Lels*) mutant in tomato revealed that the *Ls* gene is involved in the development of axillary meristems and formation of lateral buds (Schumacher et al., 1999). *OsMOC1* in rice and *AtLAS*/*SCL18* in Arabidopsis, both of which are homologous to *Lels*, were also found to play an important role in axillary meristem formation (Schumacher et al., 1999; Greb et al., 2003). *OsMOC1* is mainly expressed in the axillary buds and is required for axillary meristem initiation and tiller bud formation. *AtLAS/SCL18* is expressed in the leaf axils and its mutant does not develop axillary buds during vegetative growth in Arabidopsis (Li et al., 2003).

A large number of GRAS genes are also involved in response to biotic and abiotic stress. For example, *NtGRAS1* participates in signal transduction pathways in tobacco under stress conditions (Czikkel and Maxwell, 2007). *PeSCL7*, a GRAS family gene in *Poplar*, improves salt and drought resistance in transgenic Arabidopsis plants (Ma et al., 2010). Meanwhile, *SlGRAS6*-silenced tomato plants exhibited impaired tolerance to drought stress (Mayrose et al., 2006). Overexpression of *BrLAS*, a GRAS gene from *Brassica napus*, increased the chlorophyll content and improved drought resistance in Arabidopsis (Yang et al., 2010). Rice *OsGRAS23* is also involved in drought and oxidative stress tolerance via regulation of stress-related gene expression (Kai et al., 2015). Overexpression of the *SlGRAS40* gene in tomato was also found to enhance tolerance to abiotic stress and influence auxin and gibberellin signaling (Liu et al., 2017).

Phytohormones play pivotal roles in regulating the protective responses in plants under stress conditions. Amongst these, abscisic acid (ABA) is the one of the crucial phytohormones regulating plant growth and development under stress conditions by involving in H_2_O_2_ induced signaling (Saxena et al., 2016). H_2_O_2_, the by-product of oxidative plant aerobic metabolism, induces oxidative stress under abiotic or biotic stress conditions and acts as a secondary messenger in signal transduction networks (Jubany-Mari et al., 2009; Chen et al., 2012). The cross-talk between ABA signals and H_2_O_2_ production in plant tissue has been discussed in several reports (Pei et al., 2000; Saxena et al., 2016). ABA can induce stomatal closure by reducing the turgor of guard cells under drought stress, and H_2_O_2_, one of the major reactive oxygen species (ROS), plays a vital role as a second messenger in the ABA-induced stomatal closure (Pei et al., 2000; Miao et al., 2006). Water deficit and ABA treatment triggers H_2_O_2_ accumulation, together with major changes in gene expression of stress-responsive genes and higher antioxidant enzyme activities (Jiang and Zhang, 2002a,b). Furthermore, the increase of H_2_O_2_ content caused by water deficit was more pronounced than that response to exogenous ABA (Phillips and Ludidi, 2017). Abiotic stress condition enhances ABA/gibberellic acid (GA) ratio supporting DELLA protein accumulation which in turn lowers H_2_O_2_ levels (Considine and Foyer, 2014). Several evidences show that MAPK (mitogen activated protein kinase) cascades is implicated in ABA signaling, that is activated by the exogenous H_2_O_2_ which in turn mediated by the hormones like jasmonic acid (JA) and ABA (Rodriguez et al., 2010).

*Brassica rapa*, a member of the *Brassica* genus, is an important vegetable in China and is cultivated and consumed worldwide. However, *B. rapa* plants are frequently exposed to adverse environmental conditions, including drought, extreme temperatures, and high salinity stress. Amongst these, drought is the major stress factors resulting in reduced agricultural productivity (Abe et al., 2011; Ahmed et al., 2012). Therefore, understanding the molecular adaptation mechanisms of stress is necessary for genetic improvement of stress resistance in *B. rapa.* In this study, we isolated and characterized a GRAS transcription factor gene in *B. rapa, BrLAS*, which is an ortholog of Arabidopsis *AtLAS* (Greb et al., 2003). Expression pattern analysis revealed that *BrLAS* is expressed primarily in the roots and axillary meristems and is up-regulated under NaCl, PEG, H_2_O_2_ and exogenous ABA treatment. Overexpression of *BrLAS* also conferred pleiotropic phenotypes and enhanced drought tolerance in transgenic Arabidopsis plants. Collectively, these findings provide details of the GRAS gene family in *B. rapa*, revealing the potential function of *BrLAS* in development and drought stress tolerance.

## Materials and Methods

### Plant Materials, Growth Conditions, and Treatments

*Brassica rapa var. Japonica*, which shows multiple shoot branching during the vegetative stage, was used for *BrLAS* gene isolation and expression pattern analysis in different tissues and under various treatments. Seeds were germinated in plastic petri dishes containing two layers of filter paper saturated with distilled water at 23°C for 2 days in the dark then transferred to pots containing soil growth medium under artificial growth conditions at 23–25°C, a photoperiod of 16/8 h light/dark and relative humidity of 60 %. Hoagland nutrient solution was added once a week. Roots, stems, leaves, axillary buds and shoot tips were sampled from 4-week-old plants. Flowers were harvested after vernalization and shoot apical meristems (SAMs) were sampled every 7 days during shoot branching development (from 14 to 42 days after germination). Five well-grown plants were thoroughly mixed to form one sample. Three biological replicates of each tissue and organ were sampled.

For hormone treatment, 2-week-old seedlings were transferred to 4 L hydroponic containers containing half-strength Murashige and Skoog (1/2 MS) liquid medium (pH = 5.8) renewed once every 3 days. At the five-leaf-stage, seedlings were exposed to 1/2 MS solution with 100 μM ABA. For stress treatment, 4-week-old plants grown in pots were irrigated with 200 mM NaCl, 15 % (w/v) polyethylene glycol (PEG) and 100 mM hydrogen peroxide (H_2_O_2_), respectively. Leaves were sampled 0.5, 1, 2, 4, 8, 12, and 24 h after treatment and seedlings without treatment were used as a control. In addition, the leaves of seedlings grown under solution without any treatment were also sampled at the designated time (8:00 a.m. to 8:00 a.m. the next day). Five well-grown plants were thoroughly mixed to form one sample. Three biological replicates of each tissue and organ were sampled for RNA extraction.

*A. thaliana* ecotype Columbia (Col-0) and *BrLAS-*overexpressing Arabidopsis plants were used in this study. Seeds of transformed Arabidopsis were surface sterilized with 1% sodium hypochlorite and selected on 1/2 MS medium containing appropriate antibiotics. Homozygous lines of T_3_ generations were used for further analysis.

### Cloning of the *BrLAS* Gene

To clone *BrLAS* from *B. rapa*, a specific pair of primers, TL-F and TL-R, were designed based on sequence information in the Brassica genome database (BRAD^1^) (Wang et al., 2011) and sequence information of Arabidopsis *LAS*. The full-length cDNA of *BrLAS* was obtained via PCR using *B. rapa* total RNA, specific primers and Tks Gflex DNA Polymerase. The PCR products were then cloned into the pMD 18-T Vector (Takara, Japan) for sequencing.

*BrLAS* homologous genes were obtained by BLAST searches of the National Center for Biotechnology Information^2^ and Brassica database (BRAD, see footnote 1) using the DNA or protein sequences of *BrLAS* as queries. BrLAS-related proteins were aligned using DNAMAN software and phylogenetic relationships were analyzed using MEGA 6.0 by the neighbor-joining method with 1,000 bootstrap replicates.

### RNA Isolation and Real-Time Quantitative PCR Analysis

Total RNA was extracted using Trizol reagent (Invitrogen, Carlsbad, CA, United States) according to the manufacturer’s instructions. First-strand cDNA was synthesized from total RNA using a PrimeScript^®^RT reagent Kit (Takara). The real-time PCR reaction was performed using a Roche thermocycler (LightCycler 480, Roche, Germany). Each reverse transcription-PCR procedure was carried out with three biological and three technical replicates. The Chinese cabbage *GAPDH* gene was used as an internal control to normalize expression of the *BrLAS* gene and the relative quantification method was used to evaluate quantitative variation. ACTIN2 (At3g18780) was used as an internal control to normalize expression levels of abiotic stress-responsive genes in transgenic Arabidopsis plants.

### Constructs and Generation of Transgenic Plants

To construct the overexpression vector *35S: BrLAS*, the entire 1,332-bp coding sequence of *BrLAS* was amplified and subcloned into the pCR8/GW/TOPO entry vector (Invitrogen) according to the manufacturer’s instructions. The CDS fragment was then inserted into the pMDC32 Gateway-compatible binary vector through LR recombination reactions (Curtis and Grossniklaus, 2003). The *35S: BrLAS* plasmid was then introduced into *Agrobacterium tumefaciens* strain GV3101 and transformed into Arabidopsis Col-0 plants using the floral dip method (Clough and Bent, 1998).

### Subcellular Localization

To observe subcellular localization of the BrLAS protein, a *p35S: BrLAS-GFP* vector containing the open reading frame (ORF) of *BrLAS* (without the termination codon) fused in-frame with green fluorescent protein (GFP) and driven by the 35S promoter was constructed. The *p35S: BrLAS-GFP* and *35S: GFP* control plasmids were then introduced into GV3101 and the transformed Agrobacterium were infiltrated into leaf cells of *Nicotiana benthamiana* as described previously (Sparkes et al., 2006). GFP signals in leaf epidermal cells were subsequently observed under a Leica fluorescence microscope (Leica Microsystem, Heidelberg, Germany).

### Measurements of Chlorophyll Content

To determine the chlorophyll content, 16-day-old seedlings of wild-type (WT) and transgenic Arabidopsis grown on 1/2 MS medium were collected in centrifuge tubes and weighed then immersed in 1 mL pre-chilled 80% acetone and fully ground. After incubation at 4°C for 4 h and centrifugation at 5,000 × *g* for 10 min at 4°C, the supernatants were transferred to a 10 mL graduated tube and diluted to 3 mL with 80% acetone. A spectrophotometer was then used to measure absorbance at 645 and 663 nm, and chlorophyll a, chlorophyll b, and total chlorophyll contents were calculated as follows:

(1) $$\mathrm{Chlorophyll}\;a\;(\mathrm{mg}/\mathrm{mg})=(12.72\times A_{663}-2.59\times A_{645})\times 3/\mathrm{material}\;\mathrm{weight}(\mathrm{mg})$$

(2) $$\mathrm{Chlorophyll}\;b\;(\mathrm{mg}/\mathrm{mg})=(22.88\times A_{645}-4.67\times A_{663})\times 3/\mathrm{material}\;\mathrm{weight}(\mathrm{mg})$$

(3) $$\mathrm{Total}\;\mathrm{chlorophyll}\;(\mathrm{mg}/\mathrm{mg})=(20.29\times A_{645}+8.05\times A_{663})\times 3/\mathrm{material}\;\mathrm{weight}(\mathrm{mg})$$

### Germination and Root Growth Assay

Arabidopsis seeds of WT (Col-0) and *BrLAS*-overexpressing lines were surface sterilized with 1% sodium hypochlorite and kept at 4°C in the dark for 72 h. To conduct a germination rate assay showing the response to ABA, sterilized seeds of WT and transgenic lines were sown on 1/2 MS plates supplemented with 0 or 2 μM ABA and grown at 22°C in a growth chamber with a photoperiod of 16/8 h light/dark. Germination rates (seedling with cotyledon) were then examined after 6-day growth. For the root growth assay, seeds of transgenic and WT lines were firstly sown on 1/2 MS plates then grown at 22°C in a growth chamber with a photoperiod of 16/8 h light/dark for 5 days. Five-day-old seedlings were then transferred to fresh 1/2 MS medium supplemented with 0 or 10 μM ABA. Seedling root lengths were then measured after 8 days growth on vertical plates.

### Drought Treatment and Measurements of ABA Content

To examine drought tolerance, transgenic and WT Arabidopsis plants were germinated on 1/2 MS plates under a 16/8 h light/dark cycle at 22°C. Seven-day-old seedlings were then transferred to pots containing an identical soil mix (1:1 vermiculite:humus) under normal growth conditions. Twenty-five-day-old seedlings were then subjected to water stress by withholding watering until distinct phenotypic differences between transgenic and WT plants were observed. Plants were then re-watered and survival rates surveyed after re-watering for 3 days. To measure ABA content, leaves of WT and transgenic plants at an identical rosette stage were collected under normal and drought stress, and the procedure for extraction, purification and quantification of ABA was performed as described by Cornish and Zeevaart (1984) with some modifications. Each experiment was performed in triplicate.

### Water Loss Measurements

For water loss measurements, leaves of transgenic and WT plants at an identical rosette stage were removed and weighed immediately. The samples were then weighed at designated time intervals under normal light conditions. Leaves from five plants were mixed to form one sample. Three biological replicates were obtained for each transgenic line. Water loss was calculated as follows: (Leaf initial fresh weight - leaf weight at each time point)/leaf initial fresh weight.

### Scanning Electron Microscope (SEM)

For SEM, intact fully expanded rosette leaves of WT and transgenic plants were collected in parallel then freshly excised into thin pieces 3 mm wide and immediately fixed in 2.5% (v/v) glutaraldehyde in PBS buffer at 4°C overnight. After washing in the same phosphate buffer, the samples were post-fixed with 1% OsO_4_ and dehydrated with a graded ethanol series. After trough-critical point drying, the samples were mounted and sputter-coated with gold then observed under a SEM (HITACHI S-3400N).

### 3,3′-Diaminobenzidine (DAB) Staining

To examine H_2_O_2_ accumulation, 4-week-old rosette leaves of WT and transgenic plants under drought stress were collected and immersed in freshly prepared 1 mg mL^-1^ DAB solution for 12 h in the dark. Stained leaves were then discolored with a bleaching solution (ethanol/acetic acid/glycerol = 3:1:1) and photographed.

### Reactive Oxygen Species (ROS)-Related Biochemical Assay

To detect the changes in physiological indicators under drought stress, leaves of WT and T_3_ transgenic plants at an identical rosette stage were harvested. For extraction of H_2_O_2_, O^2-^, superoxide dismutase (SOD), peroxidase (POD) and chloramphenicol acetyltransferase (CAT), 0.2 g samples were placed in 1.8 mL 0.1 M PBS (pH 7.4) on ice. After centrifugation at 10,000 × *g* for 10 min at 4°C, the supernatants were then used to measure H_2_O_2_ and O^2-^ contents, and SOD, POD, and CAT enzyme activity using corresponding detection kits. MDA assay was performed using the thiobarbituric acid method (TBA) (Heath and Packer, 1968).

### Primers

All primers used in this study are listed in Supplementary Table S1.

### Statistical Analysis

All experiments included three independent repeats, and significant differences analyses were performed using the *t*-test at significance levels of ^∗^*P* < 0.05 and ^∗∗^*P* < 0.01.

## Results

### Cloning and Sequence Analysis of *BrLAS*

**FIGURE 1:**
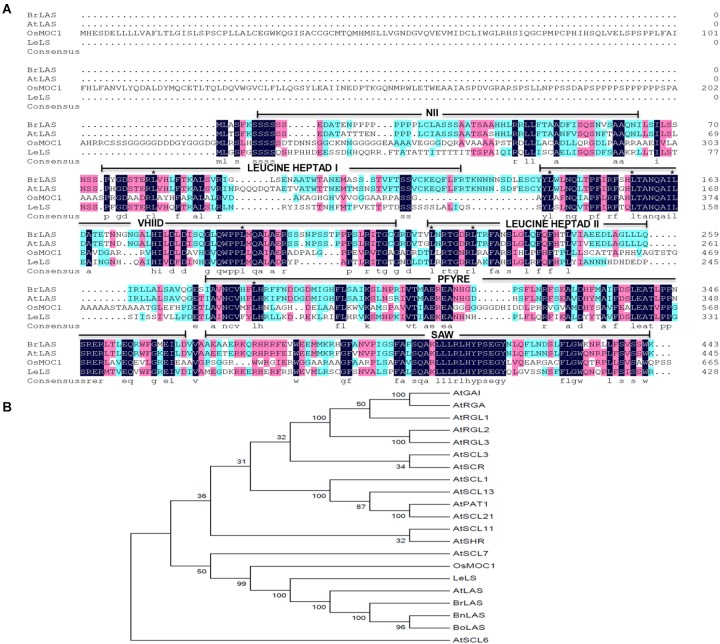
Sequence alignment and phylogenetic relationship between BrLAS and its homologous genes. **(A)** Sequence alignment of the deduced amino acid sequences of BrLAS protein, Arabidopsis AtLAS, tomato SlLS, and rice OsMOC1 as determined using DNAMAN. Identical residues are displayed by color. Conserved leucine residues in heptad repeats are identified by asterisks and conserved motifs are underlined. **(B)** Phylogenetic tree of BrLAS and its homologous proteins in *Arabidopsis thaliana, Brassica napus* (BnLAS) and *B. oleracea* (BoLAS) (GenBank accession numbers: HQ324233 and HQ324234, respectively).

The full-length cDNA sequence of *BrLAS* was 1,866-bp long and contained a 1,332-bp ORF with a 5′-untranslated region (UTR) of 151-bp and a 3′-UTR of 383-bp. It encoded a protein of 443 amino acid residues with a predicted molecular mass of 49.278 kDa. Amino acid sequence alignment analysis revealed that *BrLAS* is highly homologous to GRAS genes in other species except for its two progenitor species (*BnLAS* in *B. napus* and *BoLAS* in *B. oleracea*). For example, Arabidopsis *AtLAS* shared 89% homology, tomato *LeLs* shared 81% homology and rice *OsMOC1* shared 71% homology (Figure 1A). The deduced amino acid sequence of BrLAS contains a typical GRAS domain consisting of the LHRI, VHIID, LHRII, PFYRE, and SAW motifs in its C-terminus (Bolle, 2004) (Figure 1A). Here, phylogenetic analysis indicated that BrLAS groups with the AtLAS branch contained LeLS and OsMOC1 (Figure 1B). These results strongly suggest that *BrLAS* is a member of the GRAS gene family in *B. rapa* and an ortholog of *LAS* in Arabidopsis.

### Expression Patterns of *BrLAS*

**FIGURE 2:**
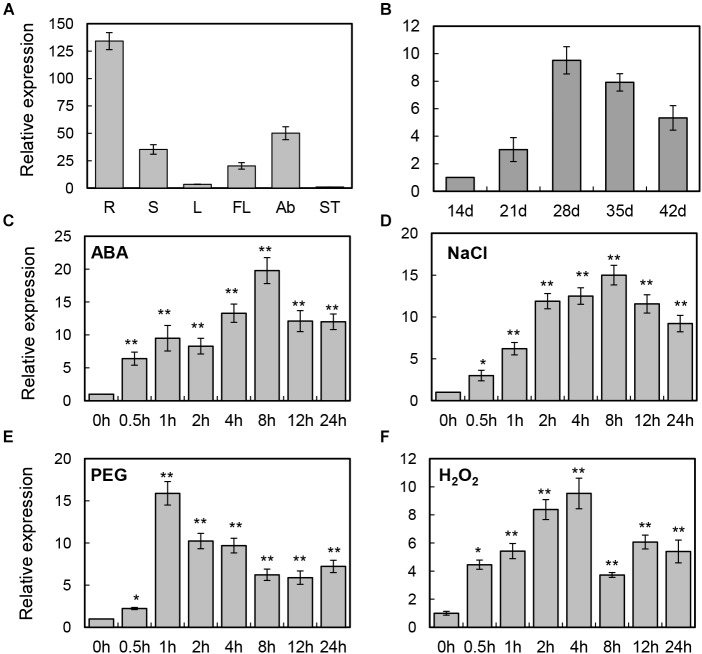
Expression patterns of *BrLAS* in *B. rapa.* **(A)** Relative expression of *BrLAS* in different tissues (R, roots; S, stem; L, leaves; FL, flowers; Ab, axillary buds; ST, shoot tips) under normal conditions. Expression data in the shoot tips were normalized to 1. **(B)** Relative expression profiles of *BrLAS* during axillary bud development (14–42 days after germination). Expression data 21 days after germination were normalized to 1. **(C–F)** Expression patterns of *BrLAS* in response to 100 μM ABA, 250 mM NaCl, 15% (w/v) polyethylene glycol (PEG), and 100 mM H_2_O_2_. Total RNA was isolated from 4-week-old seedlings exposed to the various stress treatments. Expression data of the control sample were normalized to 1. Error bars show the standard error between three replicates, asterisks indicate significant differences using Student’s *t*-test ^∗^*P* < 0.05, ^∗∗^*P* < 0.01.

To examine the potential functions of the *BrLAS* gene, quantitative real-time analysis was performed to investigate expression patterns in the roots, stems, leaves, flowers, axillary buds, and shoot tips. As shown in Figure 2A, *BrLAS* showed relatively high levels of expression in the roots, axillary buds, and flowers. Since relatively higher expression levels were observed in the axillary buds, expression levels of *BrLAS* at five stages of axillary bud development (14–42 days after germination) were examined further (Figure 2B). The results revealed that transcript levels of *BrLAS* underwent a dramatic change during axillary bus development, suggesting a role in the formation of shoot branching.

To examine the detailed expression profiles of *BrLAS* under various abiotic stresses and phytohormone treatment, 4-week-old *B. rapa* seedlings were treated with exogenous 100 μM ABA, and NaCl, PEG, and H_2_O_2_ treatment. Expression levels of *BrLAS* were significantly induced by ABA, reaching a maximum at 8 h after treatment and up-regulated more than 20-fold compared with the control, and then decreasing sharply (Figure 2C). *BrLAS* expression was also induced by NaCl, reaching a peak of more than 15-fold at 8 h after treatment (Figure 2D). Similarly, expression was markedly induced by PEG, reaching a peak of more than 15-fold at 1 h after treatment then declining gradually (Figure 2E). Under H_2_O_2_ treatment, expression reached a peak of more than 10-fold at 4 h after treatment then decreased (Figure 2F). Moreover, the expression of *BrLAS* under normal conditions using solution without any treatment was also examined, and the result showed that the expression of *BrLAS* was affected by mechanical stress, reaching a peak of approximately fivefold at 0.5 h after tissues cut then increasing gradually, and showed the circadian rhythm, the expression levels of which at 12 and 16 h (8:00 p.m. and 00:00 a.m.) were higher than other time points during the day (Supplementary Figure S1). These data suggest that expression of *BrLAS* is sensitive to NaCl, PEG, H_2_O_2_ and exogenous ABA treatment, affected by mechanical stress and showed circadian rhythm.

### *BrLAS* Is a Nuclear Protein

**FIGURE 3:**
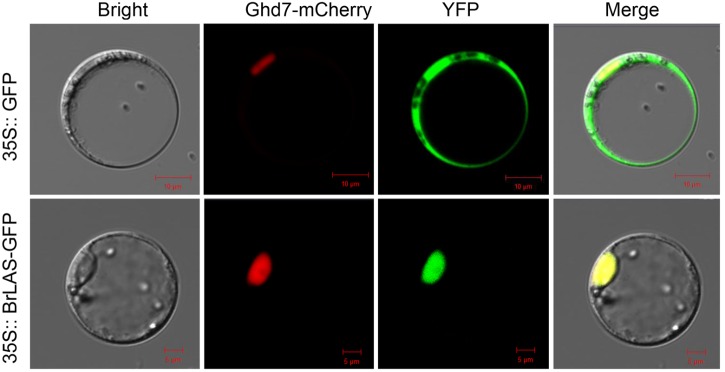
Subcellular localization of *BrLAS*. Fluorescence microscopy of *35S: BrLAS-GFP* and *35S: GFP* (control) plasmids transiently expressed in *B. rapa* protoplast cells.

To determine subcellular localization of the BrLAS protein, we constructed a *p35S: BrLAS-GFP* construct containing the ORF of *BrLAS* (without the termination codon) fused in-frame with GFP driven by the 35S promoter and transformed it into *B. rapa* protoplast cells by means of modified PEG treatment (Yoo et al., 2007). As shown in Figure 3, fluorescent signals of *35S: GFP* were uniformly distributed throughout the cells, while *BrLAS-GFP* was exclusively localized in the nucleus. Consistent with the subcellular localization of other members of the GRAS gene family and possible function as a transcription factor, these findings suggest that *BrLAS* is a nuclear protein.

### *35S: BrLAS* Transgenic Arabidopsis Plants Exhibit Pleiotropic Characteristics

**FIGURE 4:**
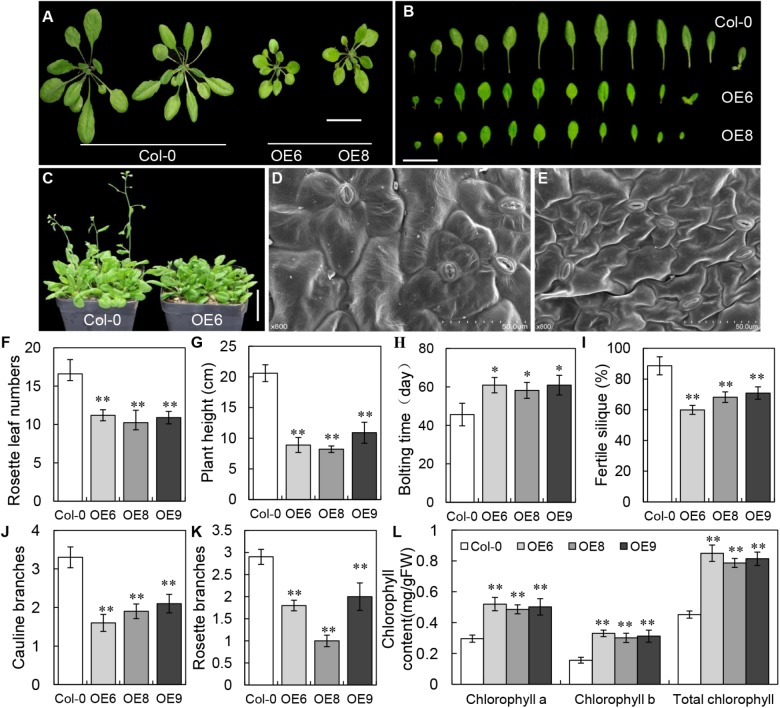
Phenotypes of *BrLAS*-overexpressing transgenic plants and wild-type (WT) plants. **(A)** Comparison of transgenic T_3_ generation OE6 and OE8 and wild-type plants. The transgenic plants were smaller and had less rosette leaves. Bar = 1 cm. **(B)** Comparison of 30-day-old rosettes leaves from WT and transgenic plants. Bar = 1 cm. **(C)** Bolting and flowering in transgenic compared with WT plants. Both were delayed in the transgenic plants. Bar = 1 cm. **(D,E)** The size of leaf epidermis cells in transgenic **(E)** and WT plants **(D)**. Cell size was significantly smaller in the transgenic plants. **(F)** Numbers of rosette leaves in the transgenic and WT plants before bolting. There were significantly less rosette leaves in the transgenic plants. **(G)** Plant height in the transgenic and WT plants at 65 days. A marked reduction was observed in the transgenic plants. **(H)** Bolting and flowering in the transgenic and WT plants. Both were delayed in the transgenic plants. **(I)** Fertility in the transgenic compared with WT plants. Reduced fertility was observed in the transgenic plants. **(J,K)** The number of rosette and cauline branches in the transgenic compared with WT plants. Both were reduced in the transgenic plants. **(L)** Chlorophyll contents in transgenic plants were increased compared with WT plants. Error bars represent the standard deviation of three independent experiments, and asterisks indicate a significant difference from the WT at ^∗^*P* < 0.05 and ^∗∗^*P* < 0.01.

To determine the potential function of *BrLAS*, we constructed a *35S: BrLAS* construct to transform Arabidopsis Col-0 plants. Approximately 40 transgenic plants were identified through hygromycin B resistance analysis and PCR verification with primers of *BrLAS* and a selection marker gene (hygromycin, HYG). Phenotypes of T_1_ transgenic plants were then observed and representative individuals were propagated for further experiments. Compared with WT Arabidopsis, the morphology and developmental characteristics of the overexpression lines showed obvious changes (Figure 4 and Supplementary Table S2). During the same developmental period, *BrLAS*-overexpressing plants grew at a slower rate and had fewer leaves, which were darker in color than the WT plants (Figures 4A,B). Some overexpressing lines exhibited delayed bolting and flowering, to varying degrees, at the reproductive stage (Figures 4C,H), with fewer senesced rosette leaves. All changes were stably inherited by the following generations; therefore, three representative T_3_ transgenic lines (OE6, OE8, and OE9) were chosen for further quantitative analysis. Rosette leaf numbers in the three overexpressing lines were significantly reduced compared to the WT plants before bolting (Figure 4F). Moreover, plant height at day 65 was markedly reduced (Figure 4G). The bolting times of all three lines were delayed by about 14 days compared with the WT (Figure 4H), and fertility was also reduced, resulting in fewer fertile siliques on the main stem (Figure 4I).

Since the majority of *BrLAS*-overexpressing plants grown both on plates and in soil showed smaller leaves with a darker green color than the WT, we also examined cell morphology and chlorophyll content. SEM showed that the leaf epidermis cells were significantly smaller in the transgenic lines than the WT (Figures 4D,E), suggesting that this was the cause of the smaller leaves in *BrLAS*-overexpressing plants. The total chlorophyll content of the three transgenic lines increased by 88.1, 74.1, and 80.1%, respectively, with increases in both chlorophyll a and chlorophyll b contents (Figure 4L), suggesting that this was the major cause of the darker green leaves in the *BrLAS*-overexpressing lines. Since the *AtLAS* gene in Arabidopsis is required for the initiation of axillary meristems, we also examined branching numbers. As shown in Figures 4J,K, numbers of rosette branches and cauline branches both decreased in the *35S: BrLAS* transgenic plants compared with the WT.

### Overexpression of *BrLAS* Confers Drought Tolerance in Transgenic Arabidopsis

**FIGURE 5:**
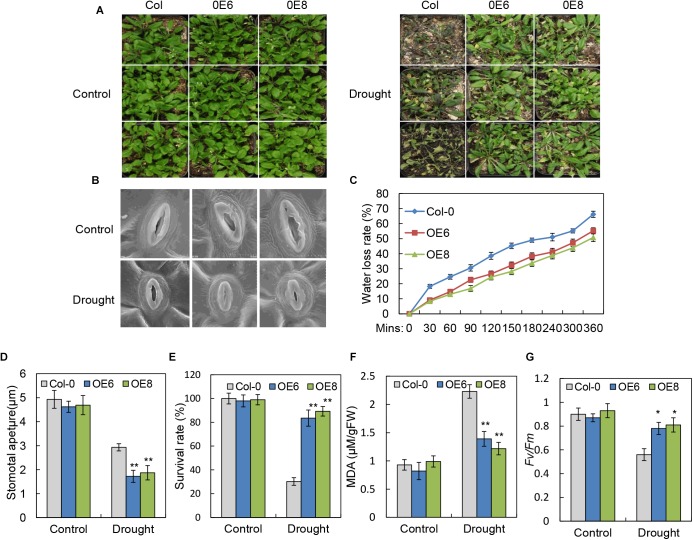
Improved drought resistance in *35S: BrLAS* transgenic Arabidopsis. **(A)** Drought resistance in *35S: BrLAS* transgenic (OE6 and OE8) compared with wild-type (WT) plants. Twenty-five-day-old seedlings grown under normal conditions were subjected to drought stress by withholding watering for up to 14 days followed by re-watering. **(B)** The stomatal apertures of *35S: BrLAS* transgenic lines compared with WT plants under drought stress. A significant decrease in size was observed in the transgenic lines. **(C)** Water loss from detached leaves of *35S: BrLAS* compared with the WT plants. A significant decrease in water loss was observed in the transgenic plants. Data represent the means of 20 leaves each per three independent experiments. **(D)** Stomatal aperture size in the *35S: BrLAS* transgenic and WT plants under drought stress. A significant decrease in size was observed in the transgenic lines. **(E)** Survival rates of the transgenic and WT plants under drought stress. **(F,G)** Comparisons of the MDA content **(F)** and *Fv/Fm* ratios **(G)** of the transgenic and WT plants. Arabidopsis seedlings treated with water were used as a control. Error bars represent the standard deviation of three independent experiments, and asterisks indicate a significant difference from the WT at ^∗^*P* < 0.05 and ^∗∗^*P* < 0.01.

Since expression of *BrLAS* was up-regulated with ABA, PEG, and H_2_O_2_ treatment (Figure 2), and considering the altered leaf morphology and delayed leaf senescence in the *35S: BrLAS* transgenic plants, it is plausible that *BrLAS* is involved in drought stress tolerance in *BrLAS-*overexpressing Arabidopsis plants. To verify this hypothesis, 3-week-old transgenic and WT seedlings were subjected to drought stress. Survival rates were then surveyed after re-watering for 3 days. As shown in Figure 5A, the leaves of WT plants displayed severe wilting and yellowing, while the overexpression lines maintained good growth after drought stress. Moreover, only 30% of WT plants were able to recover from stress, while the survival rates of the OE6 and OE8 transgenic plants were 86 and 93%, respectively (Figure 5E). Consistent with these findings, water loss rates of detached leaves from the overexpression lines decreased at each time point compared with the WT plants (Figure 5C). Stomatal apertures of WT and transgenic lines under normal and drought stress conditions were also examined. Under normal conditions, no significant differences were observed; however, at 6 h after drought stress treatment, the stomatal apertures of OE6 and OE8 leaves decreased by 65.0 and 68.4%, respectively, while those of WT plants decreased by only 35.3% (Figures 5B,D). The MDA content, a sign of oxidative damage, was also measured, revealing a significant increase in the WT plants, but only a slight increase in the transgenic plants (Figure 5F). The overexpression lines also maintained a higher *Fv/Fm* ratio than the WT plants after drought treatment (Figure 5G). These findings confirm that overexpression of *BrLAS* confers drought resistance in the transgenic plants.

### Overexpression of *BrLAS* Is Involved in the Regulation of ROS in Transgenic Arabidopsis

**FIGURE 6:**
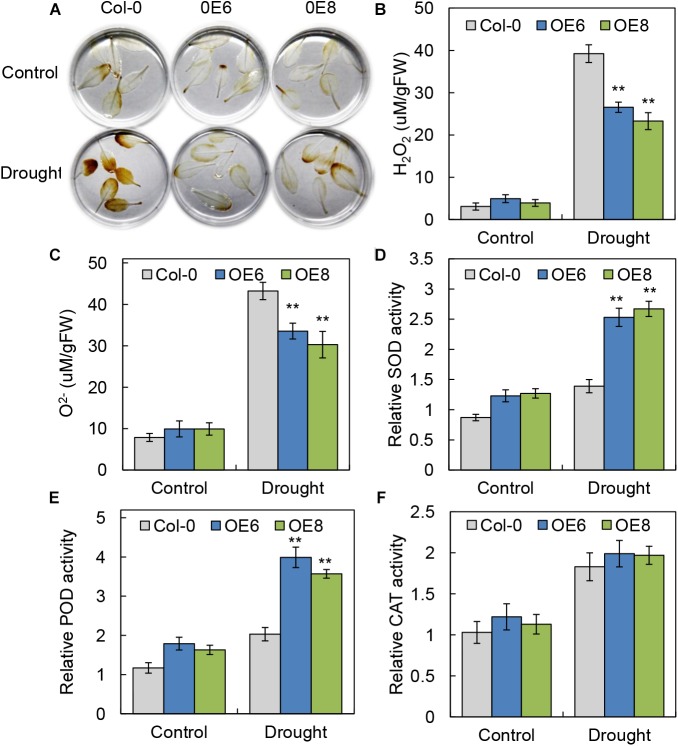
*BrLAS*-overexpressing plants exhibited increased oxidative stress tolerance. **(A)** ROS accumulation in detached leaves using DAB staining to visualize H_2_O_2_ under normal and drought stress conditions. **(B,C)** Contents of H_2_O_2_ and O^2-^ in transgenic plants and WT plants under normal and drought stress conditions. **(D–F)** Antioxidant enzyme activity in the transgenic and WT plants under normal and drought conditions. **(D)** SOD activity, **(E)** POD activity, and **(F)** CAT activity. Arabidopsis seedlings treated with water were used as a mock control. Error bars show the standard error between three replicates, and asterisks indicate significant differences from the WT at ^∗∗^*P* < 0.01.

Abiotic stress can lead to increased accumulation of ROS, having damaging effects on plant development (Wang et al., 2012). To investigate the levels of active oxygen accumulation in the transgenic and WT plants, we performed DAB staining to determine H_2_O_2_ accumulation. Compared with WT plants, the overexpression lines showed a visible decrease in DAB staining intensity under drought stress treatment (Figure 6A), consistent with the lower accumulation of H_2_O_2_ in detached leaves of the *BrLAS-*overexpressing plants. Moreover, the WT plants exhibit higher contents of H_2_O_2_ and O^2-^ than the OE lines after drought treatment (Figures 6B,C). These findings indicate that the transgenic lines experienced less damage than the WT plants under stress conditions.

Antioxidant enzyme activity is another important indicator of a plant’s ability to scavenge ROS. We therefore measured relative activities of SOD, POD, and catalase CAT (Hu et al., 2012) in WT and *BrLAS*-overexpressing lines. Activities of SOD and POD in the transgenic plants were both higher than in the WT plants under normal conditions. Meanwhile, under drought stress, levels of SOD and POD activity increased significantly in the OE lines, but only slightly in the WT (Figures 6D,E). CAT activity was up-regulated in both the OE lines and WT following drought stress treatment, with no obvious differences between the two (Figure 6F). These findings suggest that *BrLAS*-overexpression confers increased drought stress resistance in transgenic plants through enhanced ROS scavenging ability.

### *BrLAS*-Overexpressing Plants Exhibit an ABA-Hypersensitive Response

**FIGURE 7:**
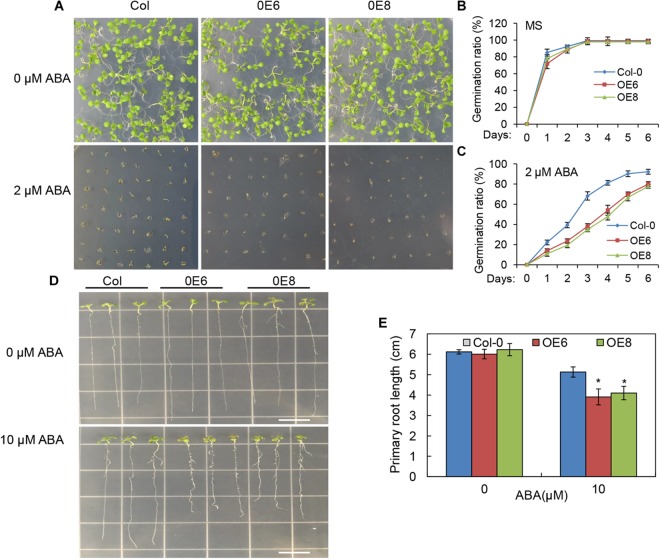
ABA hypersensitivity in the *BrLAS*-overexpressing (OE) lines. **(A)** Seed germination in wild-type (WT) and BrLAS-overexpressing lines 6 days after germination on 1/2 MS medium supplemented with 0 or 2 μM ABA and quantitative analysis of seed germination rates **(B,C)**. Values represent means ± SE (*n* = 60–90 seeds). **(D)** Phenotypes of WT and OE lines grown for 8 days on 1/2 MS medium supplemented with 0 or 10 μM ABA and primary root lengths **(E)**. Values represent means ± SE (*n* = 20–25 plants). Error bars show the standard error of three replicates, and the asterisk indicates significant differences from the WT plants at *^∗^P* < 0.05.

To determine whether the enhanced drought resistance in the *BrLAS*-overexpressing lines was associated with ABA, seeds of transgenic and WT plants were germinated on 1/2 MS medium with or without ABA. The germination rates were then compared at designated time points. No significant differences in germination rates were observed between the transgenic and WT seeds when grown without ABA (Figures 7A,B). However, on 1/2 MS medium containing 2 μM ABA, germination rates of the transgenic seeds were lower than those of WT seeds at each time point (Figures 7A,C). It is well known that ABA inhibits plant growth (Luo et al., 2014), therefore, the differences in ABA-induced inhibition of root growth were also observed. Five-day-old seedlings were transferred to 1/2 MS plates supplemented with ABA and the primary root lengths were measured after vertical growth for 8 days. Exogenous ABA-induced inhibition of root growth was more severe in the transgenic than the WT seedlings when grown on 1/2 MS medium containing 10 μM ABA (Figures 7D,E). These findings suggest that overexpression of *BrLAS* in Arabidopsis enhances plant sensitivity to exogenous ABA.

### Expression Patterns of Stress-Related Genes Under Drought Stress in Transgenic Arabidopsis

**FIGURE 8:**
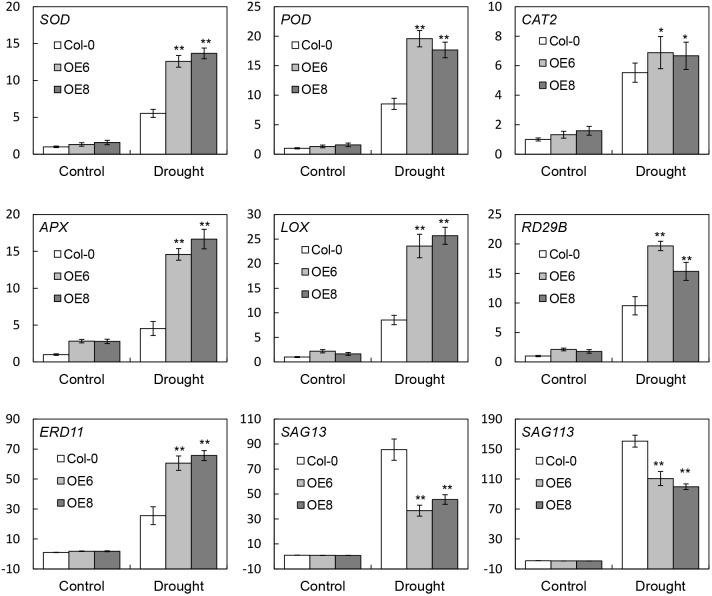
Transcript levels of stress-related marker genes in wild-type (WT) and transgenic plants under normal and drought stress conditions. Error bars show the standard error of three replicates, and asterisks indicate significant differences from the WT at ^∗^*P* < 0.05 and ^∗∗^*P* < 0.01.

To further investigate the molecular mechanism of *BrLAS*-enhanced drought stress tolerance, expression patterns of plant stress-related marker genes were examined by qRT-PCR (Figure 8). Abundant transcripts of several genes involved in ROS generation and scavenging; namely, *SOD, POD, CAT2*, ascorbate peroxidase (*APX*), and *LOX*, a lipoxygenase gene (Jin et al., 2013), were detected in *BrLAS*-overexpressing and WT plans under both normal and drought conditions. Moreover, while expression levels increased in both transgenic and WT plants, transcript levels were significantly higher in transgenic compared with WT plants under drought stress conditions, except for *CAT*. *CAT* was up-regulated in both OE and WT plants after stress treatment, with no obvious differences in expression levels, which is consistent with the change in catalase activity in both plants under drought stress (Figure 6F). Accumulation of *ERD11* (Jensen et al., 2010) and *RD29B* (Yang et al., 2011), two drought-responsive genes, was also higher in the OE than the WT plants under drought stress treatment. Meanwhile, the two leaf senescence-related genes *SAG13* (senescence-associated gene 13) (Garapati et al., 2015) and *SAG113* (senescence-associated gene 113) (Zhang et al., 2011) were both down-regulated in *BrLAS* overexpressing compared to WT plants both under normal and drought stress conditions (Figure 8), suggesting that *BrLAS* overexpression delays leaf senescence in OE plants. Taken together, these findings suggest that *BrLAS* is involved in drought stress via modulation of key stress- and senescence-related genes.

### *BrLAS*-Overexpressing Plants Had Altered Endogenous ABA Content

**FIGURE 9:**
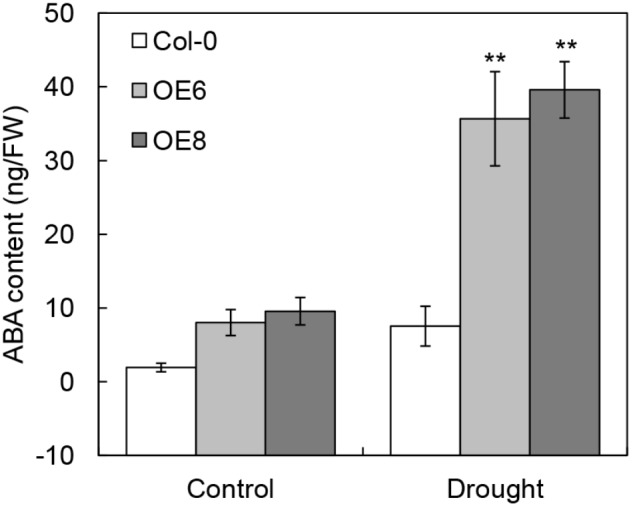
Contents of endogenous ABA in *35S: BrLAS* transgenic and wild-type (Col-0) plants under normal and drought stress conditions. Endogenous ABA contents of WT and transgenic plants were measured under normal and drought stress conditions. Error bars show the standard error of three replicates, and the asterisk indicates significant differences from the WT plants at *^∗∗^P* < 0.01.

The high-level expression patterns of ABA signaling related genes in *35S: BrLAS* plants, under normal and drought stress conditions, suggested that the endogenous ABA content may be elevated in transgenic plants. To verify this hypothesis, we measured endogenous ABA contents of transgenic and WT plants with and without drought stress. As shown in Figure 9, the ABA contents in all lines were increased under drought stress conditions. As expected, the level of ABA in *35S: BrLAS* plants were both higher than WT plants under normal and drought stress conditions. Drought stress treatment induced a dramatically increase in endogenous ABA content of *35S: BrLAS* plants, which is up to approximately fourfold higher than ABA increase in WT plants. These data demonstrate that overexpression of *BrLAS* may affect endogenous ABA content.

## Discussion

To date, available information on GRAS proteins in *B. rapa* was relatively limited, despite their important roles in plant growth and development. Song et al. (2014) analyzed the GRAS transcription factor family in Chinese cabbage and identified 48 *BraGRAS* genes in the entire genome through comparative genomic analysis. In this study, we characterized a GRAS transcription factor gene, *BrLAS*, from *B. rapa* and found that it was an ortholog of Arabidopsis *AtLAS*. *BrLAS* showed relatively high expression levels in the roots and axillary buds, and was regulated by salt, PEG and H_2_O_2_ stress as well as exogenous ABA (Figure 2). Meanwhile, transgenic *BrLAS* overexpressing Arabidopsis plants displayed significantly enhanced drought resistance with decreased ROS accumulation and increased antioxidant enzyme activity under drought treatment compared with WT plants.

*BrLAS*-overexpressing plants showed pleiotropic characteristics, including morphological changes, delayed bolting and flowering time, reduced fertility and altered chlorophyll content (Figure 4). These findings suggest that *BrLAS* is involved in various aspects of growth and development in *B. rapa*. A previous study revealed that *LAS* controls axillary meristem formation in Arabidopsis (Greb et al., 2003). Considering the high sequence identity and similar expression pattern in axillary meristems between *LAS* and *BrLAS*, it is speculated that *BrLAS* also plays a potential role in shoot branching development in *B. rapa*. Interestingly, *35S: LAS* overexpressing Arabidopsis plants not only exhibited increased shoot branches, but also presented reduced numbers of rosette and cauline branches, suggesting that relative expression levels of *BrLAS* are vital, with overexpression disrupting normal branch morphogenesis. Expression levels of *BrLAS* were also high in the roots, similar to its homologous gene *AtLAS* (Greb et al., 2003), and some *35S: BrLAS* transgenic plants showed a relatively larger number of lateral roots (Supplementary Figure S2). However, whether or not this increase in transcript abundance contributed to the increase in lateral roots in transgenic plants needs to be studied further. The darker green leaves and increased chlorophyll content of the transgenic plants was thought to be the result of smaller leaf epidermis cells, leading to more cells within a given leaf area. However, this also requires further confirmation in the future.

*BrLAS* overexpressing plants also displayed significantly enhanced drought resistance compared with WT plants (Figures 5A–G). To date, few reports have documented a role of GRAS proteins in drought stress. For example, *NtGRAS1* in tobacco (Czikkel and Maxwell, 2007), *BnLAS* in *B. napus* (Yang et al., 2010), *PeSCL7 in Poplar* (Ma et al., 2010), *OsGRAS23* in rice (Kai et al., 2015), and *SlGRAS40* in tomato (Liu et al., 2017) were found to be involved in drought stress. In this study, the increased drought tolerance in *BrLAS* overexpressing plants provides novel evidence of the role of GRAS proteins in drought stress responses. Leaf water loss rate is an important indicator of drought resistance in plants (Hu et al., 2006). Here, the *BrLAS-*overexpressing plants displayed a reduced water loss rate and smaller stomatal apertures than the WT plants (Figures 5B–D), suggesting that *BrLAS* improves water retentivity in transgenic plants by regulating stomatal closure to reduce water loss. Drought stress can also cause a decrease in photosynthetic efficiency, oxidative damage to cells, and limited metabolic reactions (Farooq et al., 2009). As an indicator of photosynthetic efficiency, *Fv/Fm* values significantly decreased in WT plants following drought treatment; however, in OE plants levels remained high, suggesting that the PSII reaction center was less damaged (Figure 5G). In addition, an increase in MDA content was observed in the WT plants, with only a slight increase in transgenic plants, suggesting that the overexpression plants suffered less oxidative damage than the WT plants (Figure 5F). On the whole, these findings suggest that *BrLAS* is involved in the regulation of drought resistance, with overexpression effectively improving drought tolerance.

Abiotic stress can trigger oxidative stress in plants (Hu et al., 2012; Wang et al., 2012), with the accumulation of ROS causing damage to plant growth. Maintaining a steady-state level of ROS within cells is important, representing balance between the amount of total reactive oxygen produced and the removal ability of the cellular antioxidant system (Foyer and Noctor, 2005). In our study, the *BrLAS-*overexpressing plants showed a significant decrease in H_2_O_2_ and O^2-^ contents compared to the WT following drought stress (Figures 6A–C). Antioxidant enzymes such as CAT, SOD, and POD also play a crucial role in scavenging ROS and enhancing stress tolerance in plants (Hu et al., 2012; Wang et al., 2012). In our study, activities of SOD and POD were both higher in the transgenic compared to WT plants under normal and drought conditions. Meanwhile, CAT activity was up-regulated in both the OE lines and WT after drought treatment (Figures 6D–F). Taken together, these findings suggest that *BrLAS* alleviated oxidative damage in the OE lines by regulating activities of antioxidant enzymes, thereby enhancing drought tolerance.

Numerous genes are reportedly up-regulated under stress conditions, with overexpression of these genes enhancing tolerance to abiotic stresses (Seki et al., 2002; Zhu, 2002). Expression levels of certain genes involved in ROS scavenging and the drought response were therefore monitored in OE and WT plants (Figure 8). Transcript levels of SOD, POD, CAT2, APX, and LOX, a lipoxygenase gene (Jin et al., 2013), were all significantly higher in OE compared to WT plants under drought treatment. Meanwhile, accumulation of *ERD11* and *RD29B*, both of which are involved in ABA-independent stress signaling pathways, was also higher in OE compared to WT plants under drought stress treatment. These findings suggest that *BrLAS* confers enhanced drought tolerance in OE plants through regulation of certain marker genes involved in abiotic stress and ROS scavenging. Moreover, expression of *SAG13* (Garapati et al., 2015) and *SAG113* (Zhang et al., 2011), senescence-related marker genes, was down-regulated in OE compared to WT plants under both control and drought stress treatment (Figure 8), suggesting that *BrLAS* leads to down-regulation of *SAG13* and *SAG113*, further contributing to enhanced drought stresses tolerance in OE plants.

Abscisic acid is a phytohormone that plays critical roles in plant stress response. It accumulates as a response to stressful environmental conditions and regulates the expression of various stress-responsive genes by a complex regulatory network (Zhu, 2002; Cutler et al., 2010; Raghavendra et al., 2010). Consistent with this function, the endogenous ABA content and expression levels of some ABA signaling related genes were changed in *35S: BrLAS* plants compared with WT plants after drought stress. It is reported that overproduction of ABA induces H_2_O_2_ accumulation, which in turn, results in the up-regulation of antioxidant enzyme activities in ABA signaling through activating MAPK cascade (Pei et al., 2000; Jiang and Zhang, 2002a,b; Zhang et al., 2007). In our study, the contents of endogenous ABA and H_2_O_2_ were both higher in *35S: BrLAS* plant (Figure 6) than WT plant without water deficit. This result indicated that overexpression of the *BrLAS* increases the cellular ABA content, which leads to the increased generation of H_2_O_2_. In contrast, the content of H_2_O_2_ in transgenic plants was lower than WT plants with the increase of ABA content under drought stress conditions. This observation may be explained at least in part by increased antioxidant enzyme activities triggered by ABA accumulation (Fath et al., 2001). Thus, our findings illustrate the complex interaction of H_2_O_2_ and ABA signaling in the regulation of drought stress response in plants and a clear integration and crosstalk between ROS and hormone signaling pathways remain to be characterized in the further research.

## Conclusion

In conclusion, this study revealed, for the first time, that *BrLAS* encodes a drought-responsive GRAS transcription factor in *B. rapa*, which was also the first identified GRAS gene positively regulating drought tolerance in leafy vegetable crops that consuming a large amount of fresh water resources in China. Compared with GRAS members in other crops, *BrLAS* is primarily involved in ABA-mediated signaling pathways, and *BrLAS*-overexpressing Arabidopsis plants exhibited hypersensitive to exogenous ABA and pleiotropic characteristics in vegetative and reproductive growth stages. These findings suggest that *BrLAS* is an ideal candidate gene for genetic manipulation of *B. rapa* drought stress tolerance breeding. The drought-resistant *BrLAS* may be used as genetic resources in drought-tolerant breeding via transgenic approaches; however, it needs huge cost and breeding time. The genome editing, a new technology, have the potential to generate varieties with improved drought tolerance by precise introduction of drought-resistant *BrLAS* into drought-sensitive, locally adapted elite varieties at a lower cost. In addition, the resistant alleles or haplotypes detected in *BrLAS* can be used as molecular markers for marker-assisted selection (MAS) in genetic improvement of *B. rapa* drought resistance.

## Author Contributions

SY, PL, and FZ designed the experiments. PL, TS, BZ, XX, WW, XZ, YY, and DZ carried out the experiments. PL, PRL, TS, SY, and FZ wrote the paper. All authors discussed the results and commented on the manuscript.

## Conflict of Interest Statement

The authors declare that the research was conducted in the absence of any commercial or financial relationships that could be construed as a potential conflict of interest.

## Footnotes

1 http://brassicadb.org/brad/

2 http://www.ncbi.nlm.nih.gov
