# Supplementary material for: BrLAS, a GRAS Transcription Factor From Brassica rapa, Is Involved in Drought Stress Tolerance in Transgenic Arabidopsis
Source: Front Plant Sci. 2018 Dec 6;9:1792. doi: 10.3389/fpls.2018.01792 (PMC6291521; doi:10.3389/fpls.2018.01792)
Supplement: Supplementary file 1 [file Data_Sheet_1.pdf]

Supplementary Material

BrLAS, a *GRAS* Transcription Factor from *Brassica rapa*, is Involved in Drought Stress Tolerance in Transgenic *Arabidopsis*

Pan Li, Bin Zhang, Tongbing Su, Peirong Li, Xiaoyun Xin, Weihong Wang, Xiuyun Zhao; Yangjun Yu, Deshuang Zhang, Shuancang Yu*, and Fenglan Zhang*

***Correspondence:** Shuancang Yu, yushuancang@nercv.org

Fenglan Zhang, zhangfenglan@nercv.org

Supplementary Table S1. Primers used in this study.

Supplementary Table S2. Comparisons of the phenotypes of wild-type and *BrLAS*-overexpressing plants.

Supplementary Figure S1 The expression profile of *BrLAS* under normal conditions in *B.rapa*.

Supplementary Figure S2. Lateral root growth in *35S: BrLAS* transgenic compared to wild-type (Col-0) plants.

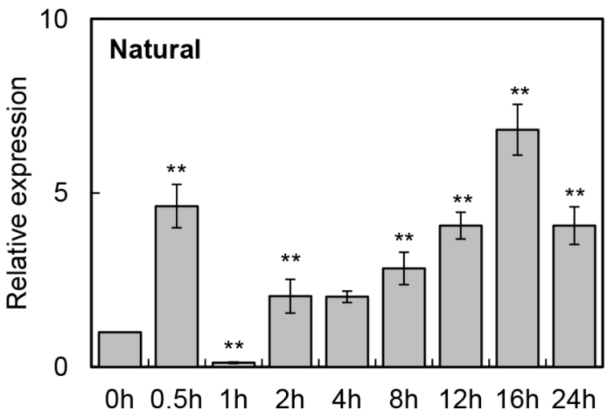

Supplementary Figure S1 The expression profile of *BrLAS* under normal conditions in *B.rapa*. The expression patterns of *BrLAS* under normal conditions using solution without ABA, NaCl, PEG, or H_2O_2. Expression data of the 0h sample was normalized to 1

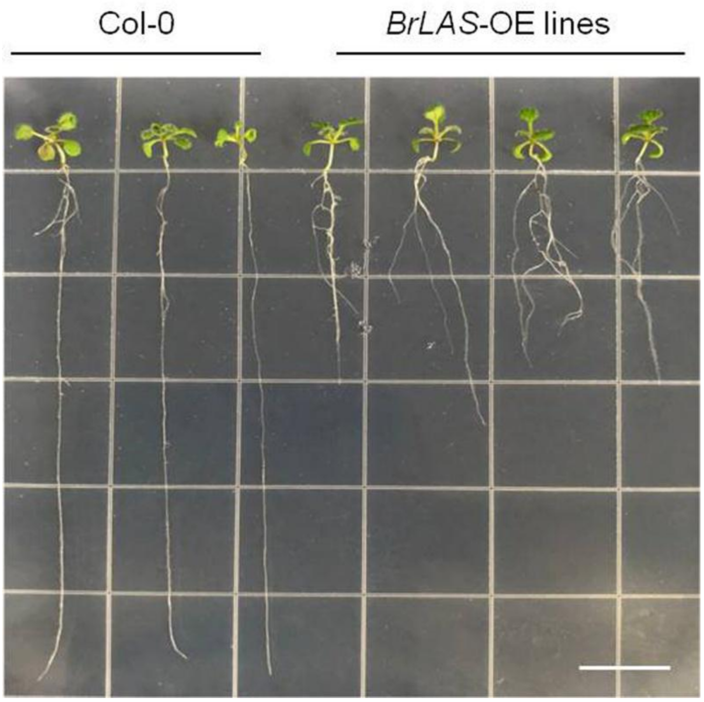

Supplementary Figure S2 Lateral root growth in *35S: BrLAS* transgenic compared to wild-type (Col-0) plants. A relatively larger number of lateral roots were observed in the transgenic compared to wild-type plants.
