# Supplementary material for: BrLAS, a GRAS Transcription Factor From Brassica rapa, Is Involved in Drought Stress Tolerance in Transgenic Arabidopsis
Source: Front Plant Sci. 2018 Dec 6;9:1792. doi: 10.3389/fpls.2018.01792 (PMC6291521; doi:10.3389/fpls.2018.01792)
Supplement: Supplementary file 2 [file Table_1.docx]

**Table S1.** Primers used in this study

| Primer name | Forward/reverse primers | | | Description | | |
| --- | --- | --- | --- | --- | --- | --- |
| **For cloning full length cDNA of *BrLAS* from B. Japonica** | |  | |  | | |
| TL-F | 5’-CTAAAGCTAGTGTTGATTTTA-3’ | | | full length cDNA cloning | | |
| TL-R | 5’-AAAATACATTTGGATCTGAT-3’ | | |  | | |
| **Primers used for RT-qPCR of *BrLAS*** | |  | |  | | |
| *BrLAS* | 5’-CATAACCGGATGTGGTCG-3’ | | |  | | |
|  | 5’-AAGTGGACGCAGTTGACG-3’ | | |  | | |
| *GAPDH* | 5’-CAGGTTTGGAATTGTCGAGG-3’ | | | Actin | | |
|  | 5’-GAGCTGTGGAAGCACCTTTC-3’ | | |  | | |
| **Primers used for** **subcellular localization of BrLAS** | | |  |  | | |
| BrLAS | 5’-ATGCTTGCTT CCTTCAAATC-3’ | | | GFP vector | | |
|  | 5’-TTTCCACGATGAAACGGAG-3’ | | |  | | |
| **Primers used for expression pattern of relevant genes in WT and *BrLAS* transgenic lines** | | | | |  |  |
| *ACTIN2* | 5'- GGTAACATTGTGCTCAGTGGTGG-3' | | |  | | |
|  | 5'- AACGACCTTAATCTTCATGCTGC-3' | | |  | | |
| *SOD* (AT1G08830) | 5'-ATGGTGTGACCACTGTGAGTG-3' | | |  | | |
|  | 5'-TCCTAGATCACCAGCATGT-3' | | |  | | |
| *POD* (AT1G14540) | 5'-CCATCCGATCTTCAATCCGA-3' | | |  | | |
|  | 5'-AAATCCTCTTGCCGATTGA-3' | | |  | | |
| *CAT2* (AT4G35090) | 5'-GAGCGTGTGGTTCATGCCAG-3' | | |  | | |
|  | 5'-CAAGGTCTCGGGACTTCCA-3' | | |  | | |
| *APX* (AT1G07890) | 5'-AACTTTCGATTGTCAATCAA-3' | | |  | | |
|  | 5'-CTGATGGAAATCAGCAAA-3' | | |  | | |
| *LOX* (AT1G55020) | 5'- TTATCTTCCACATGAAACAC-3' | | |  | | |
|  | 5'- TACTCCTGTGTCCCTCCAA-3' | | |  | | |
| *RD29B*(At5g52300) | 5'- GGAGTTCAAGATTCTGGGAAC-3' | | |  | | |
|  | 5'- CATCAAAGTTCACAAACAGAGGC-3' | | |  | | |
| *ERD11* (AT1G02930) | 5'-AGTTCTCATC GCTCTTCAC-3' | | |  | | |
|  | 5'-TGAGCTATGTATTGAGTAAT-3' | | |  | | |
| *SAG13* (AT2G29350) | 5'- AGGGAGCATCGTGCTCATATCC-3' | | |  | | |
|  | 5'- CCAGCTGATTCATGGCTCCTTTG-3' | | |  | | |
| *SAG113* (AT5G59220 ) | 5'- ATTTTCTTATTCTCGCAAGTGAC-3' | | |  | | |
|  | 5'- AAACACATTCGAACGACGCTA-3' | | |  | | |
