# Supplementary material for: BrLAS, a GRAS Transcription Factor From Brassica rapa, Is Involved in Drought Stress Tolerance in Transgenic Arabidopsis
Source: Front Plant Sci. 2018 Dec 6;9:1792. doi: 10.3389/fpls.2018.01792 (PMC6291521; doi:10.3389/fpls.2018.01792)
Supplement: Supplementary file 3 [file Table_2.docx]

**Supplementary Table S2.** Comparisons of the phenotypes of wild-type and *BrLAS*-overexpressing plants.

| Parameter | WT | OE6 | OE8 | 0E9 |
| --- | --- | --- | --- | --- |
| Rosette leaf numbers (one month after germiantion) | 14.4±0.7 | 11.8±0.6** | 10.2±1.9** | 9.3±0.9** |
| Plant height (65 day after germination , cm) | 20.6±1.4 | 8.89±2.1** | 8.2±1.2** | 10.89±1.6** |
| Bolting time (day) | 45.6±2.0 | 60.8±0.6* | 58.2±1.0* | 60.89±0.7* |
| Fertile siliques on the main stem | 88.6±1.8 | 59.89±2.6** | 68.2±3.0** | 70.89±2.7** |
| Senescence leaves(40 day after germination) | 3.0±1.1 | 1.4±0.3* | `1.2±0.6* | 0. 9±0.7* |
| Cauline branches | 3.3±0.23 | 1.6±0.2** | 1.9±0.2** | 2.1±0.2** |
| Rosette branches | 2.9±0.2 | 1.8±0.2** | 1.0±0.1** | 1.5±0.3** |

Note: Data are means ± standard deviation (30 plants). Asterisks indicate significant differences between transgenic line and wild type plants (**P < 0.05*, ***P < 0.01*)
